# Contribution of Glucosylsphingosine (Lyso-Gb1) to Treatment Decisions in Patients with Gaucher Disease

**DOI:** 10.3390/ijms24043945

**Published:** 2023-02-15

**Authors:** Tama Dinur, Peter Bauer, Christian Beetz, Claudia Cozma, Michal Becker-Cohen, Majdolen Istaiti, Arndt Rolfs, Volha Skrahina, Ari Zimran, Shoshana Revel-Vilk

**Affiliations:** 1 Gaucher Unit, Shaare Zedek Medical Center, Jerusalem 9103102, Israel; 2 Centogene GmbH, 18055 Rostock, Germany; 3 Medical Faculty, University of Rostock, 18051 Rostock, Germany; 4 Arcensus GmbH, 18055 Rostock, Germany; 5 Faculty of Medicine, Hebrew University of Jerusalem, Jerusalem 9112002, Israel

**Keywords:** glucosylsphingosine, lyso-Gb1, Gaucher disease, enzyme replacement therapy (ERT), substrate reduction therapy (SRT)

## Abstract

Glucosylsphingosine (lyso-Gb1), the deacylated form of glucocerebroside, was shown to be the most specific and sensitive biomarker for diagnosing Gaucher disease (GD). The aim of this study is to assess the contribution of lyso-Gb1 at the time of diagnosis for treatment decisions in naïve patients with GD. Newly diagnosed patients from July 2014 to November 2022 were included in this retrospective cohort study. The diagnosis was done by sending a dry blood spot (DBS) sample for *GBA1* molecular sequencing and lyso-Gb1 quantification. Treatment decisions were based on symptoms, signs, and routine laboratory tests. We diagnosed 97 patients (41 males), both type 1 (*n* = 87), and neuronopathic (*n* = 10). The median (range) age at diagnosis was 22 (1–78), with 36 children. In 65 patients, GD-specific therapy was started with a median (range) lyso-Gb1, 337 (60–1340) ng/mL, significantly higher than in patients who did not go on to treatment, 153.5 (9–442) ng/mL. Using a receiver operating characteristic (ROC) analysis, a cutoff of lyso-Gb1 > 250 ng/mL was associated with treatment with a sensitivity of 71% and specificity of 87.5%. Predictors of treatment were thrombocytopenia, anemia, and elevated lyso-Gb1 (>250 ng/mL). In conclusion, lyso-Gb1 levels contribute to the medical decision related to the initiation of treatment, mainly among mildly affected newly diagnosed patients. For patients with a severe phenotype, as for all patients, the main value of lyso-Gb1 would be to monitor response to therapy. The variable methodology and differences in the units of lyso-Gb1 measurements between laboratories prevent the adaptation of the exact cut-off we found in general practice. However, the concept is that a significant elevation, i.e., a several-fold increase from the diagnostic lyso-Gb1 cutoff, is related to a more severe phenotype and, accordingly, to the decision regarding the initiation of GD-specific therapy.

## 1. Introduction

Gaucher disease (GD), one of the most common lysosomal storage disorders, is caused by a mutant lysosomal enzyme β-glucocerebrosidase, leading to the storage of glucocerebroside. Over 860 variants within the glucocerebrosidase gene (*GBA1*) contribute to a broad spectrum of phenotypes from a perinatal-lethal to an asymptomatic form, traditionally subdivided into three main types [1]. The non-neuronopathic/adult form, type 1, is most often characterized by the presence of organomegaly (spleen and liver), hematologic abnormalities (anemia and thrombocytopenia), bone involvement, and a lack of primary central nervous system involvement [1]. The acute neuronopathic/infantile form (type 2) and the subacute/juvenile form (type 3) are both characterized by the additional presence of primary neurologic disease.

Despite being a rare disease, several therapeutic options are available for patients with type 1 GD. Three formulations of enzyme replacement therapy (ERT), all administered intravenously, usually once every two weeks, and two oral compounds of substrate reduction therapy (SRT) have been approved by the United States Food and Drug Administration (US FDA), European Medicines Agency (EMA), and major regulatory agencies [2]. In addition, there are a few imiglucerase biosimilars [3]. In 2018, we delineated our approach to managing newly diagnosed patients with type 1 GD in the era of choices [4], with two algorithms for treatment decisions: one for adults and the second for children. The algorithms highlight the fact that not all patients diagnosed with GD need to be treated and that treatment decisions should be based on clinical phenotype.

Glucosylsphingosine (lyso-Gb1), the deacylated form of glucocerebroside, was shown to be the most specific and sensitive biomarker for the diagnosis of GD [5,6]. Further research has demonstrated the utility of lyso-Gb1 as a reliable response biomarker to ERT and SRT [7,8,9]. We recently published our experience in measurements of lyso-Gb1 from dried blood spot (DBS) specimens, followed by molecular testing, for the diagnosis of GD [10]. In the present study, we evaluate how the lyso-Gb1 at diagnosis of GD may be used for management decisions in naïve patients.

## 2. Results

### 2.1. Study Cohort

Ninety-seven consequently newly diagnosed patients were included in the study (Table 1). Most patients were diagnosed with type 1 GD. The age of diagnosis varied, and approximately 40% were children (<18 years of age) at the time of diagnosis. Patients diagnosed with type 1 due to mild *GBA1* variants were significantly older, with higher hemoglobin levels and lower lyso-Gb1 compared to those with type 1 due to severe *GBA1* variants and those with neuronopathic GD (Table 1).

Gaucher disease-specific therapy, i.e., ERT or SRT, was started in 65 patients at a median (range) of 2 (0–78) months after the time of diagnosis. Six of the 32 untreated patients were not started on treatment due to personal reasons, and two were lost to follow-up. Those cases were excluded from further analysis, leaving 24 untreated patients. Starting treatment was not associated with sex or age at diagnosis. Treatment was more commonly started in patients diagnosed with type 1 GD due to severe *GBA1* variants and neuronopathic GD (Table 2).

### 2.2. Lyso-Gb1 Levels

**Figure 1:**
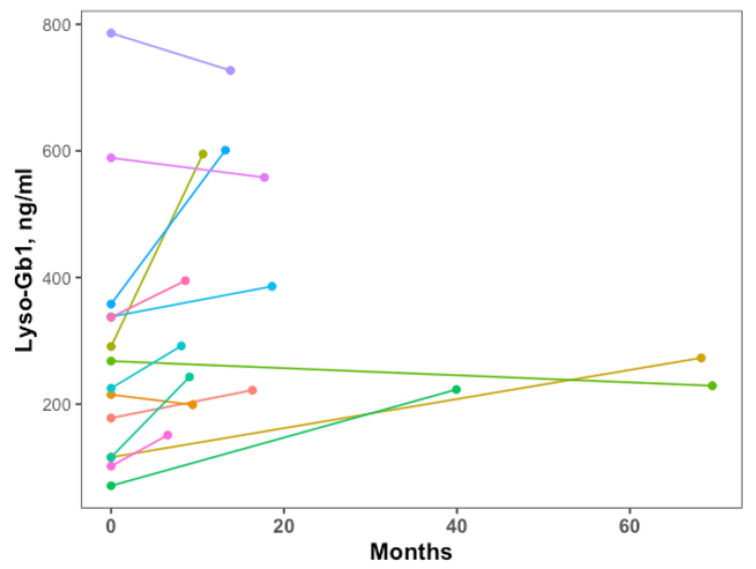
Change in lyso-Gb1 levels from diagnosis to time of initiation of therapy for patients with therapy delay (>4 months from diagnosis). The first point, time 0, is the time of diagnosis. The second point is at the time of the initiation of therapy. The colors show different subjects without any specificity.

For the 24 patients not needing GD-specific therapy during a median (range) follow-up of 3.75 (1.5–8.5) years, lyso-Gb1 at diagnosis was significantly lower compared to those starting treatment (Table 2). Of those treated, 16 patients (5 children) started therapy more than four months after diagnosis, median (range) of 11.9 (4.4–69.5) months, due to changes in disease phenotype. Changes in the lyso-Gb1 levels prior to starting are presented in Figure 1. In two cases, lyso-Gb1 was not repeated prior to therapy initiation; thus, lines are missing.

**Figure 2:**
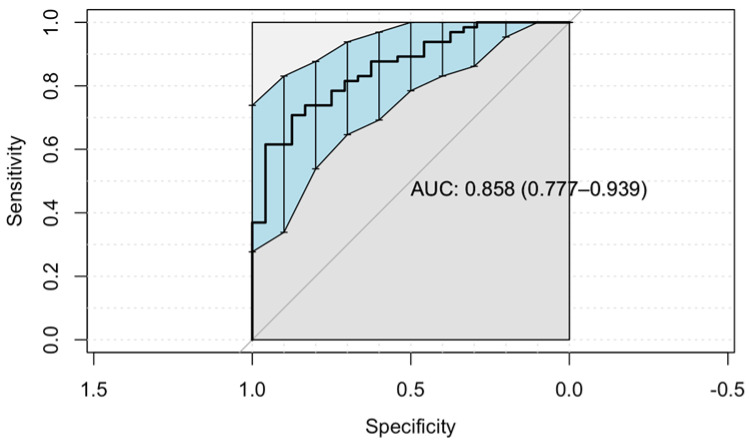
The receiver operating characteristic (ROC) curve was used to evaluate the lyso-Gb1 ability to classify initiating treatment vs. no treatment. The area under the curve (AUC) was calculated.

In order to determine the optimum lyso-Gb1 cut-off point for treatment initiation, we computed a receiver operating characteristic (ROC) curve using the “Youden” method (Figure 2). ROC curves are used to show in a graphic way the trade-off between the sensitivity and specificity for every cut-off of a diagnostic test. The cutoff found in our data was 253 ng/mL (sensitivity 71%, 95% CI 58%–81%). The specificity of this cutoff was 87.5% (95% CI 68%–97%). The area under the curve (AUC) score shows how efficient the model is; the higher the AUC, the better the model’s performance in distinguishing between the positive and negative classes. The AUC achieved in our model is considered good [11]. When including all those recommended for treatment and coding them as treated, the optimal cutoff of lyso-Gb1 remains 253 ng/mL with a sensitivity of 74% (95% CI 62%–83%) and no change in specificity.

### 2.3. Predictors for Starting Therapy

All patients with a platelet count < 100 × 10^9^/L were treated, except for two. One child had a platelet count of 50 × 10^9^/L but was otherwise asymptomatic (lyso-Gb1 37.5 ng/mL). A blood smear revealed platelet clumps. We estimated his platelet count to be >100 × 10^9^/L. Another patient had a platelet count of 86 × 10^9^/L and lyso-Gb1 > 250 ng/mL but was otherwise asymptomatic. Both had a mild genotype, i.e., homozygous for the N370S variant.

All patients with hemoglobin < 11.5 g/dL were treated, except one child with hemoglobin 10.6 due to iron deficiency anemia.

All patients with lyso-Gb1 > 250 ng/mL were treated, except four (three children) with a mild phenotype. Two adults with lyso-Gb1 < 100 ng/mL initiated therapy due to thrombocytopenia with pregnancy-related bleeding and thrombocytopenia with extensive splenomegaly. One child presenting with lyso-Gb1 < 100 ng/mL started ERT three years after diagnosis for short stature after being unresponsive to growth hormone and an increase in lyso-Gb1 to over 200 ng/mL.

Using the likelihood ratio test, the best-fitting model included platelet count, hemoglobin levels, and lyso-Gb1 levels at diagnosis. With the aim of enabling a more practical model, we transformed the continuous variables into categorical ones. Anemia was defined as hemoglobin <11.5 ng/mL and thrombocytopenia was defined as platelet count <100 × 10^9^/L. For lyso-Gb1 levels, we chose three categories: <100 ng/mL, between 100–250 ng/mL, and >250 ng/mL. The odd ratio (OR) with 95% CI for predicting treatment is presented in Table 3.

## 3. Discussion

In our study, we aimed to learn how lyso-Gb1 can contribute to treatment decisions in patients with GD by following a large cohort of patients newly diagnosed with GD, recording their treatment status, and their lyso-Gb1 levels. Although the lyso-Gb1 levels were significantly higher in patients who started treatment vs. those who remained untreated, it was not the only predictor for initiating therapy. High lyso-Gb1, i.e., several fold increase from the diagnostic cutoff lyso-Gb1 levels, was associated with the initiation of therapy.

Thrombocytopenia and anemia are well-recognized hematological manifestations of GD which, when clinically significant, warrant the initiation of therapy. However, not all cases of anemia and thrombocytopenia in patients with GD are related to GD, and isolated cytopenia without other GD-related signs and symptoms should lead to further investigation [12]. Pseudothrombocytopenia, as seen by one child, can be caused by platelet clumping in vitro and may be induced by antibody-mediated agglutination, e.g., ethylene-diamine-tetra-acetic acid (EDTA)-dependent agglutination, or aggregation secondary to platelet activation, that was found to be more common in GD [13]. The examination of a peripheral blood smear is recommended in suspected cases.

Based on our study, we believe that lyso-Gb1 levels should play an important role in treatment decisions. In contrast to the other biomarkers described in GD [14], lyso-Gb1 was shown to be associated with the pathogenesis of GD in an animal model [15]. With lyso-Gb1 accumulation, the mice model developed hematological symptoms such as reduced hemoglobin, splenomegaly, and inflammatory tissue response. Thus, relying on treatment decisions for lyso-Gb1 is important not only for its role as a biomarker but also for its role in pathogenesis.

According to the Centogene laboratory cutoff values, lyso-Gb1 levels above 6.8 ng/mL suggest GD [16]. In our recent study, all patients with GD had levels above 9 ng/mL [10]. Still, our study found a much higher lyso-Gb1 level (>250 ng/mL), predictive of initiating therapy. Notably, the fact that different laboratories are using variable methodologies for the analysis of lyso-Gb1 and that different units of lyso-Gb1 measurements are presented prevents the adaptation of the exact cut-off we found in general practice. However, one should consider the concept that a significant elevation, e.g., a several-fold increase from the diagnostic lyso-Gb1 level, is the level that is related to more severe disease and, accordingly, to the decision regarding the initiation of GD-specific therapy. This issue of variability in lyso-Gb1 measurements would hopefully be resolved, as standardization of lyso-Gb1 measurement using DBS is currently one of the missions of the International Working Group for Gaucher Disease (IWGGD) Laboratory Working Group [17].

Interestingly, while the patients that were diagnosed with genetically severe disease, i.e., severe *GBA1* genotypes and neuronopathic GD, were significantly younger compared to those with milder *GBA1* genotypes, initiation of therapy was not associated with age. Therapy was started only for symptomatic children. Asymptomatic children may grow into asymptomatic adults who also would not need therapy [18], but some may develop symptoms with time, emphasizing the importance of follow-up [4]. Although it is recommended to follow lyso-Gb1 levels in children [19], we and others have seen a spontaneous decrease in lyso-Gb1 levels in untreated children [20] that may be explained by the impact of the circadian rhythm, nutrition, physical activity, or effects of coexisting pathological conditions [21]. Thus, a one-time elevation in lyso-Gb1 may not be an indication to start therapy, while a progressive elevation in lyso-Gb1 levels would be.

Newborn screening programs for lysosomal diseases, including GD, are being developed worldwide, showing good feasibility [22,23,24,25]. These programs highlight the ethical complexity of managing infants with a later-onset disease with variable phenotype [26]. Clear guidelines on how to follow and when to start therapy for infants diagnosed with GD through screening are extremely important. The role of following lyso-Gb1 levels at this setting to identify those at risk for irreversible complications and thus benefit from the initiation of GD-specific therapy would also need to be studied.

Initiation of therapy was not associated with sex. Our observation that ERT can prevent pregnancy-related complications [27] led us, in recent years, to start ERT even in mildly symptomatic women. It would be interesting to re-assess, in several years, whether this policy change was reflected in the association between therapy and sex.

Reviewing a consecutive cohort of patients who started therapy in the last eight years prompted us to update the 1998 Israeli MOH criteria for approval of ERT [28]. The update should consider lyso-Gb1 as a key biomarker and the growing number of available GD-specific treatment options (Table 4).

In the updated criteria, we have highlighted several important points. First, lyso-Gb1 levels are important mainly for patients with milder symptoms, asymptomatic patients with a family history of significant disease, and asymptomatic patients with severe genotypes. For symptomatic cases, therapy should start irrespective of lyso-Gb1 levels. Second, age by itself is not a criterion. Third, we added a new criterion for patients with GD needing myelosuppressive therapy who would benefit from GD-specific treatment due to the significant risk of infection and/or bleeding. Due to anecdotal cases of patients with GD and monoclonal gammopathy of unknown significance (MGUS) or multiple myeloma who responded to ERT/SRT [29,30,31], the decision to treat those patients if they are otherwise asymptomatic should be made on a case-by-case basis.

Patients with type 1 GD and carriers of *GBA1* mutations have a higher propensity to develop Parkinson's disease [32]. The currently approved therapies for GD, i.e., ERT and SRT, have no role in treating or preventing Parkinson's disease in patients with type 1 GD. Thus, the diagnosis of Parkinson disease in an asymptomatic patient with type 1 GD is not considered an indication for the initiation of GD-specific therapy, especially if the lyso-Gb1 levels are not high.

## 4. Materials and Methods

### 4.1. Study Cohort

Newly diagnosed patients from July 2014 to November 2022 at the Gaucher Unit, Shaare Zedek Medical Center, were included in this retrospective cohort study. The diagnosis was done by sending a dry blood spot (DBS) sample for GBA1 molecular sequencing and lyso-Gb1 level. All samples were analyzed in Centogene GmbH, Rostock, Germany, as previously described [10]. Clinical and laboratory data, done as part of the routine assessment, were extracted from the electronic patient files. The definitions of mild versus severe genotypes were determined for type 1 GD by N370S (c.1226A > G) homozygous, and N370S/R496H (c.1604G) compound heterozygous were categorized as “mild”, whereas all other genotypes were categorized as “severe”. All treatment decisions were based on symptoms, signs, and routine laboratory tests per the Israeli Ministry of Health (MHO) criteria [28].

### 4.2. Statistical Analysis

We used the median (range) for continuous variables to report summary descriptive statistics. For nominal data, we reported the absolute and relative frequencies. The Kruskal–Wallis H test was used to determine statistically significant differences between two or more groups of an independent variable on a continuous dependent variable. A chi-square test was used to compare categorical data. The optimal.cutpoints r program with the “Youden” method was used to calculate the optimal cut point for starting treatment and plotting the ROC curves. The AUC under the ROC curve results are considered excellent for AUC values between 0.9–1, good for AUC values between 0.8–0.9, fair for AUC values between 0.7–0.8, poor for AUC values between 0.6–0.7, and failed for AUC values between 0.5–0.6 [11]. A logistic regression model was constructed to evaluate the predictors for initiating treatment using the following variables: age at diagnosis, sex, type of GD (mild type 1 GD, severe type 1 GD, and neuronopathic GD) and hemoglobin levels, platelet count, and lyso-Gb1 levels at diagnosis. We used the likelihood ratio test to compare the goodness of fit of nested regression models, and the best performing regression model was selected. A two-sided significance level of α = 0.05 was considered.

## 5. Conclusions

Our study proved the important role of lyso-Gb1 levels in the treatment decisions of patients with GD. The contribution is mainly among mildly affected newly diagnosed patients, i.e., initiation of therapy when the levels are highly elevated or rapidly progressive. The challenge of initiating GD-specific therapy in pre-symptomatic children is going to become even more relevant when newborn screening for lysosomal diseases becomes widely used, and the dependency on predictive biomarkers will be all the more critical.

## Figures and Tables

**Table 1:** Characteristics of the newly diagnosed patients.

	Type 1- Mild *GBA1* Genotype(s)	Type 1- Severe *GBA1* Genotype(s)	Neuronopathic (Type 2/3/3c)	*p*
***n***	58	29	10	
**Male, *n* (%)**	23, 39.7%	13, 44.8%	3, 30%	0.7
**Age, years: median (range)**	27.5 (2–78)	11 (1–42)	2.5 (1–25)	<0.001
**Children, %**	13, 22.4%	19, 65.5%	30, 46.2%	<0.001
**Platelet count, ×10^9^/L: median (range)**	114 (48–297)	125 (56–245)	98 (41–257)	0.7
**Hemoglobin level, g/dL: median (range)**	12.15 (9.4–15.8)	11.4 (7.1–15.5)	8.95 (6.3–12.7)	<0.001
**Lyso-Gb1, ng/mL: median (range)**	220 (9–786)	447 (102–1340)	290 (136–1270)	<0.001

The Kruskal–Wallis H test was used to study the differences between the two groups. The definition of mild vs. severe *GBA1* genotypes is detailed in the methods.

**Table 2:** Characteristics of treated and untreated patients.

	GD-Specific Treatment		
	No	Yes	*p*
***n***	24 *	65	
**Male, *n* (%)**	11, 45.8%	24, 38%	0.68
**Age, years: median (range)**	26.5 (1–78)	21 (1–71)	0.29
**Children, %**	9, 37.5%	30, 46.2%	0.62
**Type of Gaucher disease, %**			0.005
Type 1- mild *GBA1* genotype(s)	21, 87.5%	32, 49.2%	
Type 1- severe *GBA1* genotype(s)	2, 8.3%	24, 36.9%	
Neuronopathic (Type 2/3/3c)	1, 4.2%	9, 13.8%	
**Platelet count, ×10^9^/L: median (range)**	165 (53–297)	98 (41–257)	<0.001
**Hemoglobin level, g/dL: median (range)**	13.2 (10.6–15.8)	11.6 (6.3–15.6)	<0.001
**Lyso-Gb1, ng/mL: median (range)**	153.5 (9–442)	337 (60–1340)	<0.001

* those recommended for therapy but were unwilling or unable to start treatment were excluded. The Kruskal–Wallis H test was used to study the differences between the two groups. The definition of mild vs. severe *GBA1* genotypes is detailed in the methods.

**Table 3:** A multivariate model of predictors for initiating Gaucher disease (GD) treatment.

Variables	GD-Specific Therapy		Odd Ratio (OR) (95% CI)	*p*
	No	Yes		
**Platelet count, ×10^9^/L**				
≥ 100	22 (92%)	31 (48%)	1	
< 100	2 (8%)	34 (52%)	16.5 (3.4–143)	0.002
**Hemoglobin level, mg/dL**				
≥ 11.5	22 (92%)	36 (55.5%)	1	
< 11.5	2 (8%)	29 (44.5%)	7.5 (1.5–58)	0.02
**Lyso-Gb1 level, ng/mL**				
<100	8 (46%)	3 (5%)	1	
100–250	12 (50%)	17 (26%)	3.2 (0.5–29)	0.2
>250	4 (17%)	45 (69%)	22.8 (3.4–239)	0.003

A logistic regression model was constructed with a likelihood ratio test to compare the goodness of fit of nested regression models. The best-performing regression model was selected.

**Table 4:** Suggested updated criteria for initiation of Gaucher disease-specific treatment.

Israeli Ministry of Health Criteria for Imiglucerase, 1998 [28]	Suggested Updated Criteria for ERT/SRT
A family history in a sibling with a rapid acceleration in the course of the disease.	When symptomatic or high lyso-Gb1 *
Age of onset of signs or symptoms of the disease below 5 years of age	Age is not a criterion by itself
At any age, enlargement of the spleen and liver is accompanied by signs of hypersplenism, abnormal liver function tests, or other complications	Stays as is
Hypersplenism expressed as serious pancytopenia: hemoglobin below 9 g%, white count below 3000/mm^3^, platelet count of 50 000/mm^3^ or less, in consecutive blood tests during a three-month approximated	Gaucher-related significant, symptomatic cytopenia and/or bleeding disorder, **irrespective of lyso-Gb1 levels**
Symptomatic anemia which is not the result of iron deficiency or due to other causes unrelated to Gaucher disease, or thrombocytopenia with a tendency to bleeding, or a consistently decreasing platelet count.	Redundant
Signs of an autoimmune mechanism which are persistent or increasing during a follow-up period of a few years.	With **high lyso-Gb1**
Bone involvement expressed as recurrent or acute crises of pain, with objective documentation (e.g. MRI, bone scan, X-ray, or the expert opinion of a rheumatologist or orthopedist), spinal compression or evidence of other serious changes on X-ray even in the absence of pain.	Bone pain or evidence of significant bone involvement in MRI/ DEXA or any imaging abnormalities and the presence of **high lyso-Gb1**
Lung involvement	Stay as is
Evidence of decreased height or weight accompanied by a clinical picture of malnutrition.	Short stature after exclusion of other causes or with the presence of **high lyso-Gb1**
Molecular diagnosis of a ‘severe’ genotype (other logical parameters and of the reduction in organ- than homozygosity for N370S (1226G)) in the presence of signs or symptoms which may be milder than those stated above; however, this criterion does not include asymptomatic patients.	Molecular diagnosis of a ‘severe’ genotype ** and **high lyso-Gb1**
-	Any patient who was diagnosed with malignancy requiring myelosuppressive therapy

* High lyso-Gb1 in our cohort would be lyso-Gb1 > 250 ng/mL based on measurements performed in Centogene on DBS. However, we use the term “high lyso-Gb1” (a several-fold increase from the diagnostic lyso-Gb1 cutoff) to reflect the variability in methodology and unit of lyso-Gb1 measurements between laboratories. ** non N370S (c.1226A > G) homozygous or N370S/R496H (c.1604G) compound heterozygous.ERT, enzyme replacement therapy; SRT, substrate reduction therapy; MRI, magnetic resonance imaging; DEXA, dual-energy X-ray absorptiometry.

## Data Availability

Data cannot be shared due to ethical and privacy issues.
